# Preface to Symposium E: Nanoscaled Si, Ge based Materials

**DOI:** 10.1186/1556-276X-6-105

**Published:** 2011-01-31

**Authors:** Fabrice Gourbilleau, Artur Podhorodecki

**Affiliations:** 1 CIMAP, CNRS/CEA/ENSICAEN/UCBN Ecole Nationale Superieure d’Ingenieurs de Caen, Caen, France; 2 Laboratory of Advanced Optical Spectroscopy, Institute of Physics, Wroclaw University of Technology, Wybrzeze Wyspianskiego 27, 50-370 Wroclaw, Poland

## 

This special volume of *Nanoscale Research Letters *features selected papers presented at the Symposium E: "Nanoscaled Si, Ge based Materials" of the EMRS Fall meeting, held in Warsaw, Poland from September 13-^h^16, 2010.

The symposium was organized by Fabrice Gourbilleau (CIMAP laboratory, Caen France), Artur Podhorodecki (Wroclaw University of Technology, Institute of Physics, Poland) and Nicola Daldosso (University of Trento, Physical Department, Trento, Italy). CNANO North West (GDR 2975, CNRS, FRANCE) kindly sponsored the symposium.

The goal of the symposium was to describe exciting, state-of- the- art applications of Si or Ge nanoparticle-based materials, doped or not with rare earth ions, in the fields of (i) information storage, (ii) optoelectronic devices, (iii) telecommunications, as well as (iv) life sciences. using the. The origin of such intense research activity in these different domains can be explained by the potential of Group IV nanostructures to lead to either (i) future chips with optical interconnects in which CMOS compatible electronic and photonic and/or biosensing components aree integrated or (ii) the future generation of solar cells with lower cost and/or higher efficiency.

The four-day symposium included 13 sessions in which 48 oral and 18 poster presentations gave a comprehensive overview of progress toward a wide range of applications as well as the development of new promising techniques for microstructural investigations at the atomic scale. The papers published in this special volume have been selected after careful peer review, and form an authoritative reference for future applications.

The organizers wish to thank the EMRS staff for their help in the organization of the symposium.

